# Indications, feasibility and outcome of robotic retroperitoneal lymph node dissection for metastatic testicular germ cell tumours

**DOI:** 10.1038/s41598-021-89823-y

**Published:** 2021-05-21

**Authors:** Carsten-Henning Ohlmann, Matthias Saar, Laura-Christin Pierchalla, Miran Zangana, Alena Bonaventura, Michael Stöckle, Stefan Siemer, Julia Heinzelbecker

**Affiliations:** 1 Department of Urology, Malteser Krankenhaus Seliger Gerhard, Bonn, Rhein-Sieg Germany; 2 Department of Urology and Paediatric Urology, Saarland University Medical Centre and Saarland University Faculty of Medicine, Homburg, Saar Germany; 3 Present Address: Department of Urology, Johanniter Krankenhaus, Bonn, Germany

**Keywords:** Testicular cancer, Germ cell tumours

## Abstract

Data on robotic retroperitoneal lymph node dissection (R-RPLND) for metastatic testicular germ cell tumours (mTGCTs) are scarce and the use of R-RPLND itself is still under debate. The aim of our study was to evaluate the indications, feasibility and outcomes of R-RPLND, with special emphasis on differences between primary R-RPLND (pR-RPLND) and post-chemotherapeutic R-RPLND (pcR-RPLND) in mTGCTs. We retrospectively analysed the data of patients who underwent R-RPLND for mTGCT between November 2013 and September 2019 in two centres in Germany. Indications, operative technique, intra- and postoperative complications and oncologic outcome were analysed. Twenty-three mTGCT patients underwent R-RPLND (7 pR-RPLND, 16 pcR-RPLND). For pR-RPLND versus pcR-RPLND, median time of surgery was 243 min [interquartile range (IQR) 123–303] versus 359 min (IQR 202–440, p = 0.154) and median blood loss 100 mL (IQR 50–200) versus 275 mL (IQR 100–775, p = 0.018). Intra- and postoperative complications were more frequent in pcR-RPLND (pcR-RPLND: intra/post: 44%/44%; pR-RPLND: intra/post: 0%/29%). However, these were only statistically significant in the case of intraoperative complications (intra: p = 0.036, post: p = 0.579). Intraoperative complications (n = 7), conversions (n = 4) and transfusions (n = 4) occurred in pcR-RPLND patients only. After a median follow-up of 16.3 months (IQR 7.5–35.0) there were no recurrences or deaths. R-RPLND displays a valuable, minimally invasive treatment option in mTGCT. However, R-RPLND is challenging and pcR-RPLND especially bears a considerable risk of complications. This operation should be limited to patients with an easily accessible residual tumour mass and to surgeons experienced in robotic surgery and TGCT treatment.

## Introduction

Testicular germ cell tumours (TGCTs) affect young men, but the disease itself has an excellent cure rate^1^. Retroperitoneal lymph node dissection (RPLND) is an important column of TGCT treatment, though it has a shrinking importance in clinical stage (CS) 1^2–5^. Nevertheless, in metastatic TGCT (mTGCT), RPLND remains an integral part of treatment in both the primary (p) and post-chemotherapeutic (pc) setting^2,4^. To date, RPLND is mainly performed by open surgery, and has ever since been associated with a relatively high complication rate^6^. With the introduction of minimally invasive surgery, promising results were achieved with laparoscopic RPLND (L-RPLND)^7,8^. Due to the technical advantages of robotic surgery, several groups started to transfer the results of L-RPLND into robotic RPLND (R-RPLND)^9–12^. Since the beginning of the development of minimally invasive RPLND, concerns have been raised, mainly in terms of perioperative morbidity and oncologic outcome^7,13^. It is apparent that a minimally invasive approach can only be called successful if its advantages are not outweighed by perioperative morbidity and oncologic outcomes.

We thus evaluated the indications, feasibility and outcomes of R-RPLND in mTGCT, with special emphasis on differences between pR-RPLND and pcR-RPLND.

## Patients and methods

### Patient population

We retrospectively analysed the records of all patients who underwent R-RPLND for mTGCT between November 2013 and September 2019 at the Urology Departments of Saarland University Medical Centre, Homburg [n = 18, 11/2013–09/2019, open RPLND: n = 9 (33%)], and Malteser Hospital, Bonn, a teaching hospital of the University Hospital Bonn [n = 5, 07/2018–08/2019, open RPLND: n = 0 (0%)]. Patients were offered R-RPLND in case of limited retroperitoneal disease and when appearing technically feasible according to preoperative imaging. All patients gave written informed consent and anonymized patient data were used. This proceeding was approved by with the ethics committee of Saarland (identification number: 135/20). All methods were performed in accordance with the relevant guidelines and regulations. Each indication for R-RPLND was thoroughly approved according to guidelines, histological findings, clinical stage and patient compliance. Staging of the patients was performed according to the current TNM and IGCCCG classifications^1,14^. In pcR-RPLND patients, standard frontline chemotherapy regimens included bleomycin/etoposide/cisplatin (n = 14) or cisplatin/etoposide/ifosfamide (n = 2, due to concerns on bleomycin because of heavy smoking and/or inhaling drug abuse).

### Surgical technique

Operations were performed by five experienced surgeons (C.-H.O., S.S., J.H., M.S., M.S.) for open and robotic RPLND. C.-H.O. and S.S. took part in all operations, either as the primary surgeon or as supervisor.

The extent of RPLND depended on the primary site of metastasis. Modified RPLND template dissection was performed in patients with low-volume disease, limited to the primary landing zone of the affected testis^15–17^. The right-sided template included the right common iliac, paracaval, precaval, retrocaval, and interaortocaval lymph nodes and the right gonadal vein; the left-sided template included the left common iliac, preaortic, para-aortic, and retroaortic lymph nodes to the level of the inferior mesenteric artery, and the left gonadal vein^16^. Low volume disease in pcR-RPLND patients was defined according to the extension of lymph node diameter after chemotherapy. In all other patients a bilateral, nerve-sparing (whenever possible) template was resected.

**Figure 1:**
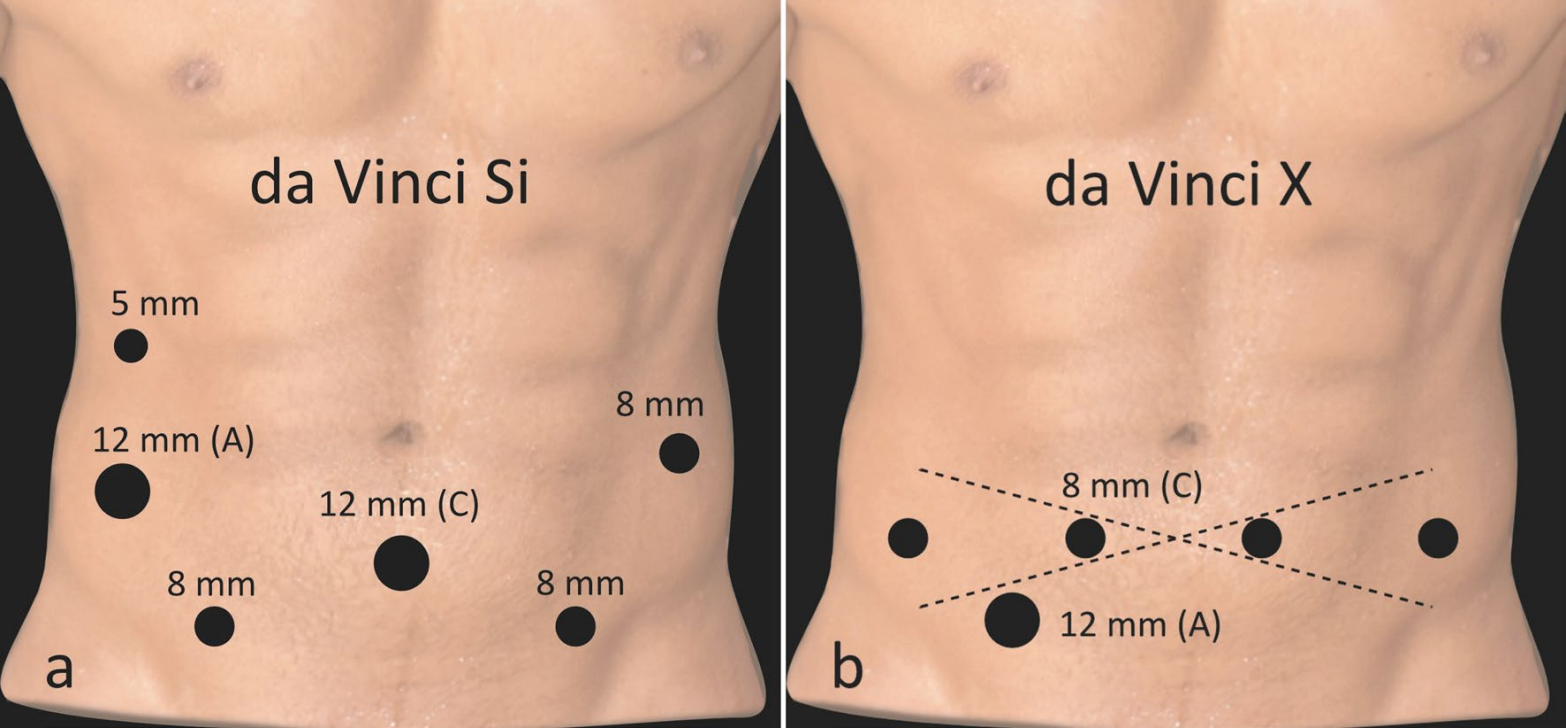
Port placement. (**a**) Da Vinci Si: Port placement included three 8-mm trocars—two above the anterior superior iliac spine on each side and a third one approximately 8 cm cranially on the left side underneath. The camera trocar (12 mm) was placed in the midline, 5 cm below the umbilicus, in line with the two 8-mm trocars. For the assistant physician, a 12- and 5-mm trocar was inserted on the right side below the costal arch. Port placement was the same for unilateral and/or bilateral cases. (**b**) Da Vinci X: Port placement included four 8-mm da Vinci trocars, including the 8-mm camera trocar (right lower abdomen), inserted in one line in the lower abdomen with equivalent distances to each other. For the assistant physician, a 12-mm trocar was inserted in the right lower abdomen, even more caudal to the da Vinci trocars. If a unilateral modified template is dissected, this approach can be modified according to Stepanian et al., with the line of the robotic trocar placement turning towards the aimed template^24^. We thank Thomas Gebhardt for creating the figure.

The da Vinci Si and X four-arm surgical systems (Intuitive Surgical, Palo Alto, California, USA) were used. A transperitoneal approach was applied. For trocar placement in the supine position see Fig. 1.

**Figure 2:**
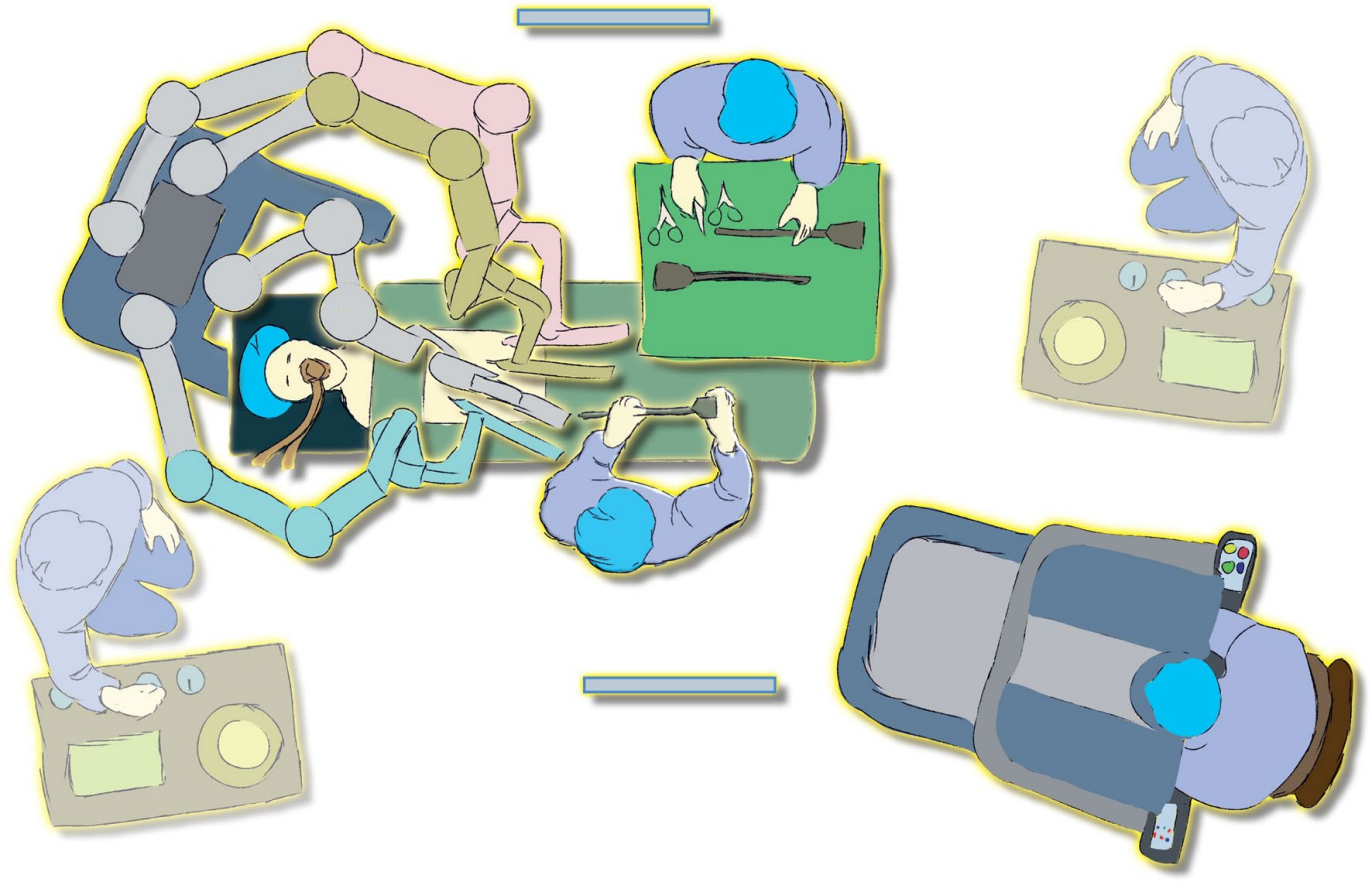
Operating room set-up for robotic retroperitoneal lymph node dissection (R-RPLND). We thank Thomas Gebhardt for creating the figure.

Afterwards, the patient was placed with the head downward and the legs slightly bent. In addition, the left side was rotated 15° downwards, with the system docked from the head of the patient over the left shoulder (Fig. 2).

Access to the retroperitoneum started with the mobilisation of the caecum and ileum by incision of the peritoneum. Thus, the colon and small intestines fell away from the operative field. With the third robotic arm the peritoneum was retracted. If necessary—i.e., in obese patients or at poor exposure—percutaneous stay sutures were placed to keep away the peritoneum^18^. After identification of the dissection field margins, the lymph nodes (LNs) were removed by the split-and-roll technique, starting from the caudal border at the crossing of the iliac artery and ureter^19^. Dissection then continued cranially with the precaval and pre-aortal LNs. Inter-aortocaval LNs were mobilised, starting at the aortic bifurcation, where the sympathetic nerve fibres of the hypogastric plexus were identified and secured to achieve a nerve-sparing technique. After mobilisation of the descending colon, it was retracted by the assistant port. Paraaortic LNs above the inferior mesenteric artery (IMA) were easily accessed from the right side. Ligation and division of the IMA were only needed in selected cases. Access to the retrocaval and retroaortic LNs was achieved using vessel loops and a 30° optical lens. Dissected LN tissue of specified resection areas was removed using the 12-mm assistant port or non-permeable retrieval bags.

### Assessment of complications and Follow-up

Intra- and postoperative complications were defined as any variation from the usual course. Postoperative complications were assessed using the Clavien–Dindo classification system^20^. Patient follow-up was performed according to the recommendations of the European association of Urology^2^. Relapse was defined as elevation of tumour markers or progressive disease on imaging.

### Statistical analysis

Continuous variables are shown as medians with interquartile ranges (IQR), and categorial variables were assessed using frequencies and proportions. Differences between groups were calculated with the Mann–Whitney U test for continuous variables and with the Chi^2^ test for categorial variables. A *p*-value of < 0.05 was considered statistically significant. Statistical analysis was carried out using SPSS 25 (IBM Corporation, Armonk, New York, USA).

## Results

### Baseline patient characteristics

A total of 23 mTGCT patients underwent R-RPLND. Thirteen were performed using the da Vinci Si platform and ten were performed using the X platform. Seven (30%) received pR-RPLND and 16 (70%) received pcR-RPLND. For baseline and preoperative characteristics, see Table 1.

**Table 1:** Baseline characteristics of patients prior to R-RPLND.

Patients n = 23	pR-RPLND n = 7	pcR-RPLND n = 16
Median age, year (IQR)	43 (23–46)	32 (26–45)
Median BMI, kg/m^2^ (IQR)	27.2 (19.9–35.3)	27.8 (23.4–31.8)
**ASA Score, n (%)**		
I	3 (43)	5 (31)
II	4 (57)	10 (63)
III	0 (0)	1 (6)
IV, V	0 (0)	0 (0)
**Primary site of TGCT**		
Left	3 (43)	10 (63)
Right	4 (57)	6 (38)
**Primary tumour histology, n (%)**		
NSGCT	4 (57)	14 (88)
Seminoma parts	5 (71)	8 (50)
Teratoma parts	4 (57)	9 (56)
Yolk sac tumour parts	2 (29)	7 (44)
Embryonal cell carcinoma parts	2 (29)	13 (81)
Chorioncarcinoma parts	0 (0)	3 (19)
Seminoma	3 (43)^a^	2 (13)^b^
**Clinical stage, n (%)**		
2A^c^	7 (100)	1 (6)
2B	0 (0)	8 (50)
2C	0 (0)	3 (19)
3A	0 (0)	1 (6)
3B	0 (0)	2 (13)
3C	0 (0)	1 (6)
**IGCCCG classification, n (%)**		
Good	7 (100)	9 (56)
Intermediate	0 (0)	5 (31)
Poor	0 (0)	2 (13)
Median largest tumour diameter at R-RPLND, cm (IQR)	1.7 (1.4–1.9)	2.3 (1.8–4.0)

*yr* years, *IQR* interquartile range, *BMI* body mass index, *ASA* American Society of Anaesthesiologists, *TGCT* testicular germ cell tumour, *NSGCT* nonseminomatous germ cell tumour, *IGCCCG* International Germ cell Cancer Collaborative Group, *R-RPLND* robotic retroperitoneal lymph node dissection, *pR-RPLND* primary robotic retroperitoneal lymph node dissection, *pcR-RPLND* post-chemotherapeutic robotic retroperitoneal lymph node dissection.

^a^Two seminoma patients received pR-RPLND in CS 2A and one patient in CS 2A at relapse under surveillance. They either denied radiotherapy/chemotherapy or R-RPLND was performed to gain diagnostic accuracy. The patient at relapse in CS 2A was upgraded to CS 2B (pN2) disease after R-RPLND. Of the two other patients only one had a vital seminoma metastasis (CS 2A, pN1), the other patient was downgraded to CS 1 disease. None of them received further adjuvant treatment and all of them are recurrent free and alive at a median follow-up of 12 (IQR 10–18) months.

^b^Two seminoma patients received pcR-RPLND: one trisomy 21 patient because of life-threatening intolerance to chemotherapy in CS 2B with only two cycles of chemotherapy being administrable and one in CS 2C with haemodynamic relevant compression of the inferior caval vein due to the residual tumour mass. In both cases histology revealed necrosis only.

^c^One NSGCT patient presented with relapse in the retroperitoneum of less than 2 cm 5.2 years after diagnosis of CS 1S disease treated initially with three cycles of chemotherapy. This patient was comprised in the CS 2A and pcR-RPLND patients. Final histology revealed a teratoma and was classified as ypN2 disease.

Most of the patients were nonseminomatous TGCT (NSGCT) patients (78%), predominantly CS 2 (83%) with a good prognosis (70%). The main indication for R-RPLND in mTGCT was pc residual mass. All patients had normalised tumour markers (AFP, β-HCG, LDH) prior to R-RPLND.

Two seminoma patients underwent pcR-RPLND; one due to life-threatening recurrent septic conditions during the first two of three planned cycles of chemotherapy, most probably associated to trisomy 21 (CS 2B patient)^21^; the other one due to a haemodynamic relevant compression of the inferior caval vein by the residual tumour (CS 2C).

Three patients relapsed under surveillance in CS 1, one seminoma and two NSGCT patients. Two of them (one seminoma, one NSGCT) relapsed with CS IIA (both marker negative) and were treated with pR-RPLND; the third patient (NSGCT) relapsed with CS IIB and was treated with chemotherapy. Because of residual tumour disease, he underwent pcR-RPLND afterwards. Three seminoma patients received pR-RPLND in CS 2A, two at primary diagnosis and one at relapse under surveillance. One NSGCT patient presented with late relapse at 5.2 years after chemotherapy for CS 1S disease with a less-than-2-cm mass in the retroperitoneum. All were marker-negative at R-RPLND.

### Perioperative outcome

In most of the patients, a unilateral modified template was performed (n = 14 (61%)). The median tumour diameter at R-RPLND was 1.9 cm (IQR 1.7–3.3). Fifteen patients (65%) underwent nerve-sparing procedures. For perioperative outcomes, see Table 2.

**Table 2:** Peri- and postoperative patient outcome.

Patients n (%)	Overall 23 (100)	pR-RPLND 7 (30)	pcR-RPLND 16 (70)	p-value
Median operation time, min (IQR)	303 (195–435)	243 (123–303)	359 (202–440)	0.154
Median EBL, ml (IQR)	200 (100–450)	100 (50–200)	275 (100–775)	0.018*
**Resection template, n (%)**				
Bilateral	8 (35)	0 (0)	8 (50)	0.039*
Unilateral modified	14 (61)	7 (100)	7 (44)	
Lumpectomy^a^	1 (4)	0 (0)	1 (6)	
**Nerve-sparing, n (%)**				0.172
Yes	15 (65)	6 (86)	9 (56)	
No	8 (35)	1 (14)	7 (44)	
**Median nodal yield, n (IQR)**	11 (7–26)	11 (6–17)	11 (7–27)	0.49
Bilateral	26 (12–30)	na	26 (12–30)	
Unilateral modified	12 (8–27)	11 (6–17)	8 (8–16)	
Lumpectomy^a^	1 (na)	na	1 (na)	
**Lesion histology, n (%)**				ne
Necrosis or normal tissue	8 (25)	2 (29)	6 (38)	
Teratoma	6 (26)	1 (14)	5 (31)	
Germ cell tumour	9 (39)	4 (57)	5 (31)	
**Final nodal stage, n (%)**				0.298
pN0	8 (35)	2 (29)	6 (28)	
pN1	7 (30)	1 (14)	6 (38)	
pN2	8 (35)	4 (57)	4 (25)	
pN3	0 (0)	0 (0)	0 (0)	
**Complication rate, n (%)**				
Intraoperative	7 (30)	0 (0)	7 (44)	0.036*
Postoperative^b^	9 (39)	2 (29)	7 (44)	0.493
**Intraoperative complications**				
Bleeding lumbar artery	1	na	1	
Partial resection of ureter	1	na	1	
Partial resection of ileum	1	na	1	
Poor exposure	2 (→ conversion)	na	2 (→ conversion)	
Ventilation problems	1 (→ conversion)	na	1 (→ conversion)	
Bleeding renal vein, nephrectomy	1 (→ conversion)	na	1 (→ conversion)	
**Clavien-Dindo Classification**^**b**^**, n (%)**				
I	0	0	0	
II bleeding with blood transfusion^c^	4	0	4	
IIIa chylous ascites	1	1	0	
lymphocele	4	1	3	
IIIb wound dehiscence^d^	1	0	1	
Compartment syndrome left leg^d^	1	0	1	
Chylous ascites	1	0	1	
IVa	0	0	0	
IVb	0	0	0	
Conversion rate, n (%)	4 (17)	0 (0)	4 (25)	0.146
Transfusion rate, n (%)	4 (17)	0 (0)	4 (25)	0.146
Length of stay, days (IQR)	6 (4–9)	4 (4–9)	6 (5–10)	0.376
Median follow-up, months (IQR)	16.3 (7.5–35.0)	11.5 (7.0–27.4)	19.2 (7.9–35.0)	0.492

*pR-RPLND* primary robotic retroperitoneal lymph node dissection, *pcR-RPLND* post-chemotherapeutic robotic retroperitoneal lymph node dissection, *n* patient number, *IQR* interquartile range, *EBL* estimated blood loss, *na* not applicable, *ne* not evaluated.

*Statistically significant p < 0.05.

^a^Lumpectomy was performed in one patient with hemodynamic relevant compression of the caval vein by residual tumour of a seminoma.

^b^Complications within 90 d postoperatively according to Clavien-Dindo Classification.

^c^Three of them converted patients.

^d^Converted patient.

Median operation time was 303 min (IQR 195–435) in all cases, with 243 min (IQR 123–303) and 359 min (IQR 202–440) for pR-RPLND and pcR-RPLND (p = 0.154). Median estimated blood loss was 200 mL (IQR 100–450), being significantly higher in pcR-RPLND, with 275 mL (IQR 100–775) compared to 100 mL (IQR 50–200) in pR-RPLND (p = 0.018). Four patients (17%) received blood transfusions (all pcR-RPLND).

Intraoperative complications occurred only in pcR-RPLND patients (n = 7, 30% compared to n = 0, 0%) (p = 0.036). Of them, three were successfully managed robotically—bleeding of a lumbar artery (without indication for transfusion), partial resection of the left ureter by performing an end-to-end anastomosis and partial resection of the ileum by establishing an end-to-end anastomosis. Ureteral injury was due to tumour involvement and bowel injury occurred iatrogenic due to thermal injury. In four cases, conversion to open surgery was decided upon. Adhesion of tumour tissue to the renal vein was managed by nephrectomy to prevent further blood loss and enabling complete resection of residual tumour tissue. In two of the first patients, poor exposure of the retroperitoneum led to conversion. In another patient with large-volume CS 3A disease, anaesthetic ventilation problems marked by subsequently rising pulmonary pressure due to the pneumoperitoneum forced late conversion after prolonged operation time.

Postoperative complications occurred in nine patients (39%), of whom seven (30%) had major complications (Clavien–Dindo ≥ III; Table 1)—two (29%, all major) patients of the pR-RPLND and seven (44%, five major) of the pcR-RPLND group (p = 0.493). The most frequent major complications were chylous ascites and lymphoceles. Most of them could be managed by placing a drainage under local anaesthesia. However, in one patient chylascites had to be treated by open surgical ligation of the thoracic duct. In two other patients, grade IIIb complications occurred—a secondary wound dehiscence in one and a compartment syndrome of the left lower leg in another patient, both managed with surgery. Median length of stay was 6 days (IQR 4–9), with four days (IQR 4–9) in the pR-RPLND and 6 days (IQR 5–10) in the pcR-RPLND patients (p = 0.376).

### Oncologic outcome and follow-up data

On pathological review, the median nodal yield was 11 (IQR 7–26); 26 (IQR 12–30) in bilateral and 12 (8–27) in modified unilateral resected templates. The most frequent histology was germ cell tumour (GCT) in nine (39%) patients, with GCT (n = 4, 57%) being the most frequent histology type in the pR-RPLND and necrosis (n = 6, 38%) being the most frequent in the pcR-RPLND patients. Eight patients (35%) had no metastatic lesions (pN0), seven (30%) were classified as pN1 and eight (35%) were classified as pN2. In pR-RPLND patients, two (29%) were downgraded to pathological stage (PS) 1 disease, one seminoma and one NSGCT patient. Four (57%) of the pR-RPLND patients were upgraded from CS 2A to PS 2B (pN2) disease, two seminoma and two NSGCT patients (R-RPLND: teratoma; embryonal cell carcinoma). In none of the cases was adjuvant treatment performed. All patients had negative postoperative tumour markers. At a median follow-up of 16.3 months (IQR 7.5–35.0) there were no recurrences or deaths in any patients (see Table 2).

## Discussion

The use of minimally invasive RPLND is still under debate, as concerns regarding morbidity and oncologic efficacy exist. Compared to open RPLND, minimally invasive RPLND has the advantage of lower blood loss, less pain, shorter length of stay and cosmetic benefits^7–10,12,22^. However, laparoscopic RPLND is challenging and therefore R-RPLND, with its technical benefits, may overcome the limitations of laparoscopic RPLND.

To date, most of the studies on R-RPLND have reported on pR-RPLND^10,18,22–25^, with the largest cohort comprising 58 patients including mainly CS 1 tumours^22^. Only a few publications on pcR-RPLND are available^11,12,26,27^, with the largest series comprising 45 patients^27^. So far, only one other publication exists on R-RPLND in a mere mTGCT patient collective, comprising both pR-RPLND (n = 22) and pcR-RPLND (n = 4) patients^28^. Such reports define the possibilities of R-RPLND for mTGCT patients, taking into account the nearly vanished relevance of pR-RPLND in CS 1 patients, in whom the treatment options nowadays are surveillance or one cycle of chemotherapy in high-risk patients^2,3,5^. Additionally, no direct comparison between pR-RPLND and pcR-RPLND in mTGCT has been made. In the present study, it became evident that pR-RPLND and pcR-RPLND for mTGCT may be challenging in different ways. The results of our series of patients demonstrate the feasibility of R-RPLND for a wide range of indications, as well as the differently challenging character of pR-RPLND compared to pcR-RPLND, with acceptable morbidity and excellent early oncologic efficacy in mTGCT patients.

Positioning of the patient and trocars is crucial for optimal exposure, with poor exposure being the most frequent reason for conversions in the beginning of our series. We used an approach with the patient in the supine position. Alternative positioning included patient in the flank^10–12^ or lower lithotomy position^18^, with the limitation that only unilateral or modified template resection was possible. The positioning of the patient in the supine position, with all of the trocars placed in the lower abdomen, offers ideal access to the retroperitoneum, enabling both uni- and bilateral template resection^24^. Using this approach, R-RPLND has a broad range of indications in mTGCT, ranging from patients primarily presenting CS 2A/B disease to those at relapse under surveillance and those with residual disease after chemotherapy, all of whom were included in our analysis.

With a median estimated blood loss of 200 mL, our study demonstrates the minimally invasive nature of the technique^12,22^. A median time of surgery of 303 min—243 in pR-RPLND and 359 in pcR-RPLND—seems to be quite long, however is comparable to the results of others^12,22^.

When comparing the pR-RPLND with the pcR-RPLND patient collective, we observed a higher operating time, conversion rate, blood loss, transfusion rate and complication rate in the pcR-RPLND patients. In terms of statistical significance, only blood loss was higher and intraoperative complications occurred more frequently in the pcR-RPLND patients. A direct comparison of pR-RPLND and pcR-RPLND is not available to date. Rocco et al. reported an intra- and postoperative complication rate of 3.3% and 33%, respectively, in mainly CS 1 pR-RPLND patients^22^. This is in line with our complication rates, which were 0% for intra- and 29% for postoperative complications in the pR-RPLND group. Pc-RPLND is challenging even in open surgery, and bears the risk of intraoperative complications and additional procedures^6^. Li et al. reported an overall postoperative complication rate of 32%, including 19% major complications for both pcR-RPLND and open RPLND^12^. In total, 20% (10% major) of the complications occurred in pcR-RPLND, and 40% (24% major) in open RPLND patients. Other large open RPLND series report on 18% major postoperative complications with vascular injury being the most common intraoperative and ileus the most frequent postoperative complication^29^. We report a higher overall postoperative complication rate of 39% (30% major), comprising both pR-RPLND and pcR-RPLND. The most common major postoperative complications in our series were chylous ascites (8%) and lymphoceles (17%) that in most cases were easily managed by putting in a drainage. Large studies on open pc-RPLND report lower lymphocele rates compared to chylous ascites rates of 3% vs. 13%, with chylascites being the second most frequent general postoperative complication^29^. Thus patients should be informed about these important complications preoperatively. In our series all intraoperative complications, conversion to open surgery and major postoperative complications (Clavien–Dindo ≥ 3b) occurred during the first three years after implementation of the technique and in the pcR-RPLND collective only, illustrating that a learning curve is relevant. Thus even in a high volume robotic surgery centre (> 600 robotic procedures per year) a learning curve of R-RPLND has to be taken into account. Furthermore these results demonstrate that pcR-RPLND is more challenging than pR-RPLND. Thus, especially at the implementation of the technique, one has to bear a higher complication rate in mind, and starting in patients with limited disease seems reasonable. In summary our results are in line with the results of other large volume series on R-RPLND^22,27,28^.

Tumour size in the retroperitoneum is the most important challenging factor in open pc-RPLND^30^. This might even be more relevant for R-RPLND. According to our experience, patients with limited disease in the retroperitoneum are ideal candidates for R-RPLND. The altered desmoplastic tissue after chemotherapy and the differences in seminoma and NSGCT histology are additional challenging reasons, even in open RPLND^31^. However, although limited to two patients, our results demonstrate that pcR-RPLND in special indications is feasible even in seminoma patients.

Minimally invasive RPLND series have been criticised for incomplete template dissection. With our positioning of the patient in the supine position, all relevant template resections, even bilateral templates, are possible. Our median nodal yield was 11, 26 in bilateral and 12 in modified unilateral templates, which is lower than that described in other studies^10,12,22^. This may be due to the wide variation in indications and resected templates. Furthermore, postchemotherapeutic tumours are often large convoluted masses, making nodal count in this setting not reliable and highly dependent on the pathologists expertise^32^. Concerning pR-RPLND, there are no reliable studies on retroperitoneal nodal counts in healthy patients. Additionally, we did not perform a pathologic re-review. Traditionally, a wide variation in lymph node yields exists, depending on whether they are performed en bloc or in packages and how pathology processing and specimen evaluation are performed^32^.

The results reported by Calaway et al. should attract attention^33^. They report on five patients that were referred to their department after R-RPLND elsewhere, all presenting with recurrences in unusual locations. Reasons for this, such as poor patient selection, poor operative technique and surgical technology, are discussed. The median time to recurrence was 259 days (range 92–503). In our own series, with a median follow up of 495 days (IQR 251–933), we have so far seen no recurrences or deaths. Nevertheless, our results need to be validated with a longer follow-up period.

As indications for pR-RPLND in CS 1 disease, due to the nowadays most often applied treatment options of surveillance or one cycle of chemotherapy, have almost vanished, our study shows the wide indications for R-RPLND in mTGCT in a real-world setting^2,3,5^. Of note, several patients with special indications were included—seminoma patients in CS 2A, patients at relapse under surveillance, a patient at late relapse after chemotherapy and pc seminoma patients. Of the pR-RPLND mTGCT patients, three were seminomas in CS 2A disease. All of these patients denied radiation or chemotherapy, and with the aim of offering an approach that reduces toxicities and overtreatment and, in response to the setting of the SEMS and PRIMETEST trials, pR-RPLND was performed^34–37^. All of these patients were discharged after three to four days. None of them received adjuvant treatment and all of them are relapse free at 24, 12 and 9 months, respectively. Even though these results need confirmation with longer follow-up and in a prospective setting, they show the possibilities of pR-RPLND in mTGCT patients, holding the promise of sparing adjuvant therapies, providing excellent oncologic outcomes and at the same time offering a minimally invasive therapy approach^5,38,39^.

Concerning pc-RPLND, as reliable prognostic markers for residual disease in RPLND specimens are still lacking, we are overtreating up to 40–50% of pc TGCT patients^16^. Thus the extent of the dissection and it’s morbidity should be as limited as possible^40,41^. This can be achieved with a robot-assisted approach and a limited dissection template^41^.

There are several limitations to our study, including its retrospective character, the missing evaluation of antegrade ejaculation and the pathologic re-evaluation of the number of lymph nodes dissected. Additionally, different surgeons were involved in the operations. A larger series and a longer follow-up are needed to further determine the role of R-RPLND in mTGCT.

## Conclusion

R-RPLND offers a treatment option for mTGCT in both the primary and post-chemotherapeutic setting. PR-RPLND in mTGCT displays a minimal-invasive treatment option whilst sparing adjuvant therapies associated with mortality increasing long-term toxicities. Pc-RPLND is associated with a 40–50% chance of overtreatment. The robotic approach offers a minimal-invasive treatment alternative to these patients. PcR-RPLND is more challenging than pR-RPLND. Both, pR-RPLND as pcR-RPLND, are providing excellent oncologic outcome in the mTGCT patient setting. Carefully chosen mTGCT patients, in the hands of surgeons experienced in TGCT treatment and robotic surgery, may profit from minimally invasive R-RPLND.
